# ^1^H NMR and FT-IR dataset based structural investigation of poly(amic acid)s and polyimides from 4,4′-diaminostilbene

**DOI:** 10.1016/j.dib.2016.02.006

**Published:** 2016-02-09

**Authors:** Amit Kumar, Seiji Tateyama, Katsuaki Yasaki, Mohammad Asif Ali, Naoki Takaya, Rajeev Singh, Tatsuo Kaneko

**Affiliations:** a School of Materials Science, Japan Advanced Institute of Science and Technology (JAIST), 1-1 Asahidai, Nomi-shi, Ishikawa 923-1292, Japan; b Faculty of Life and Environmental Sciences, University of Tsukuba, Ibaraki 305-8572, Japan; c Material/Organometallics Laboratory, Department of Chemistry, ARSD College, University of Delhi, Delhi 110021, India; d JST, ALCA, Japan

## Abstract

Structural investigation of polymers by various available analytical methods is important in order to correlate the structure with polymer properties for which understanding of polymer structure is very important factor. The data presented here in this article shows the ^1^H NMR spectra used for the characterization of prepared poly(amic acid)s (PAAs). It is often difficult to assigns the peak in NMR of polymers due to its complexity. Data presented here helps in assigning the proton peak in complex NMR of PAAs prepared from aromatic diamines. Further functionality in polymer chains can be confirmed by FT-IR spectra. Change in functionality during some reaction or process can be monitored by disappearance or appearance of peaks in FT-IR. The complete imidization of PAAs to Polyimides (PIs) is difficult to analyze because of the chemical stability i.e. insolubility of PIs in most of the solvent therefore the completion of imidization process was confirmed using FTIR.

## 1 **Specifications Table**

**Table:** 

Subject area	*Chemistry*
More specific subject area	*Polymer chemistry*
Type of data	*NMR and FTIR spectra*
How data was acquired	*NMR and FT-IR.*^1^H NMR were performed on a Bruker Biospin AG 400 MHz spectrometer. FT-IR spectra were recorded with a Perkin-Elmer Spectrum One spectrometer between 4000 and 400 cm^−1^ using a diamond-attenuated total reflection (ATR).
Data format	*Analyzed*
Experimental factors	^1^H nuclear magnetic resonance (^1^H NMR) was performed using DMSO-*d*_6_ as a solvent at 23.1 °C. FT-IR of PAAs and PIs were recorded using thin films.
Experimental features	Poly(amic acid)s (PAAs) are synthesized by a reaction of diamines 4-4′ diaminostilbene (DAS) with equimolar amounts of dianhydrides by polycondensation reaction. Polyimide (PI) films were obtained by stepwise thermal imidization of the PAAs films in an oven.
Data source location	School of Materials Science, Japan Advanced Institute of Science and Technology (JAIST), 1-1 Asahidai, Nomi-shi, Ishikawa, 923-1292, Japan
36°26′40.3″N, 136°35′33.5″E
36.444528, 136.592639
Data accessibility	*Data is with this article.*

## 2 Value of the data

• This following data helps in assigning the proton peak which is otherwise a complex task in case of polymers.
• The comparison with various other polymers can help in understanding the significant role of aromatic diamines and dianhydride structure in structural-property of polymers.
• The completion of imidization can be easily confirmed by FT-IR by comparing the spectra before and after imidization process.

## 3 Data

**Fig. 1:**
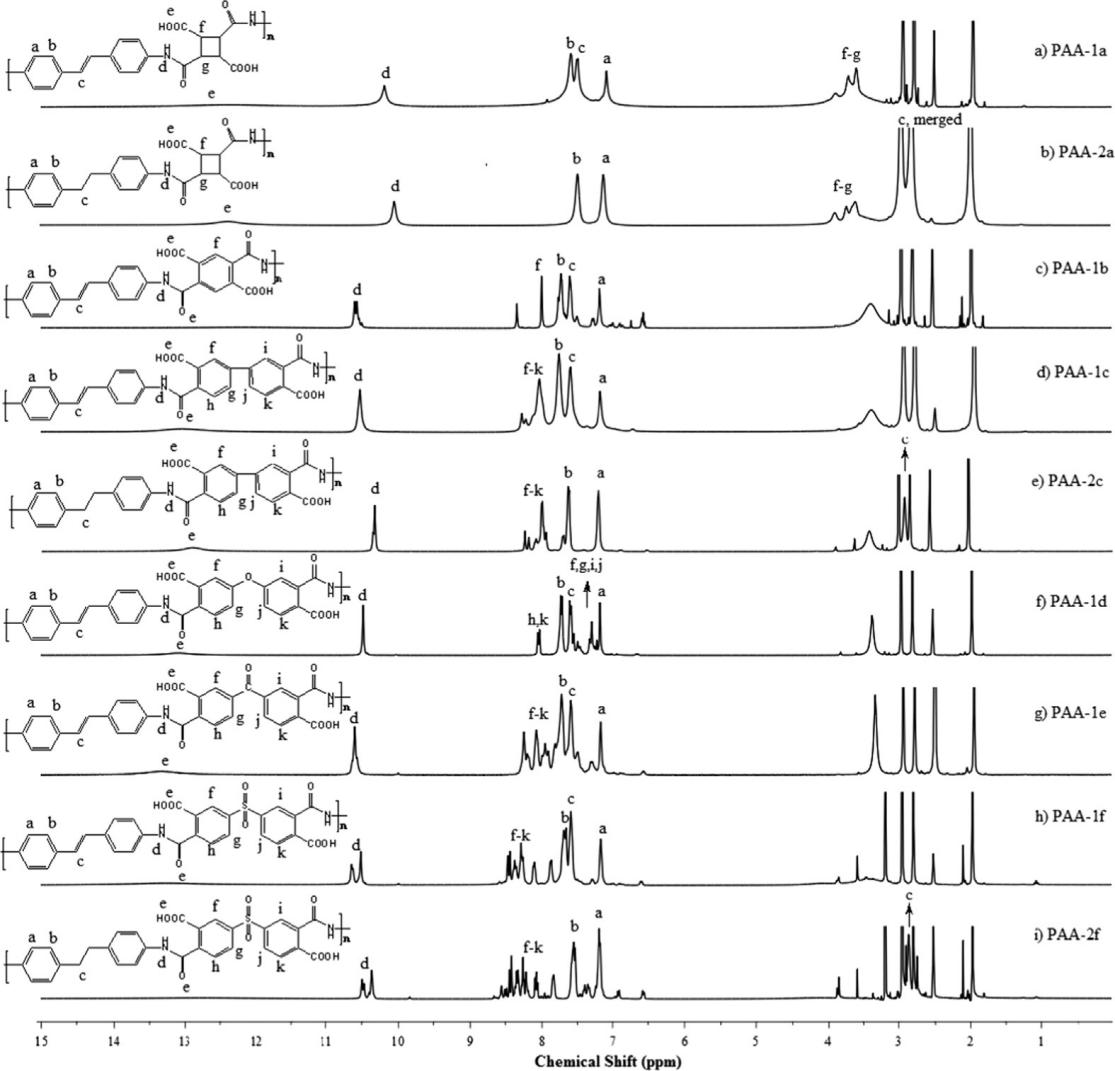
^1^H NMR spectra of (a) PAA***-***1a, (b) PAA-2a, (c) PAA-1b, (d) PAA-1c, (e) PAA-2c, (f) PAA-1d, (g) PAA-1e, (h) PAA-1f and (i) PAA-2f.

In ^1^H NMR spectra (Fig. 1) of PAAs derived with 4-4′diaminostilbene (DAS), the main chain proton signals for amides, aromatics proton of dianhydride, aromatic proton of diamine DAS and –CH=CH– appeared around 10.7–10.1, 8.6–7.3, 7.6–7.1 and 7.6–7.4 ppm respectively. In case of 4,4′-(ethane-1,2-diyl)dianiline (EDDA) derived PAAs, –CH=CH– protons peaks was replaced by proton peak of –CH_2_–CH_2_– at 2.8–2.7 ppm however other peaks remained unaltered.

**Fig. 2:**
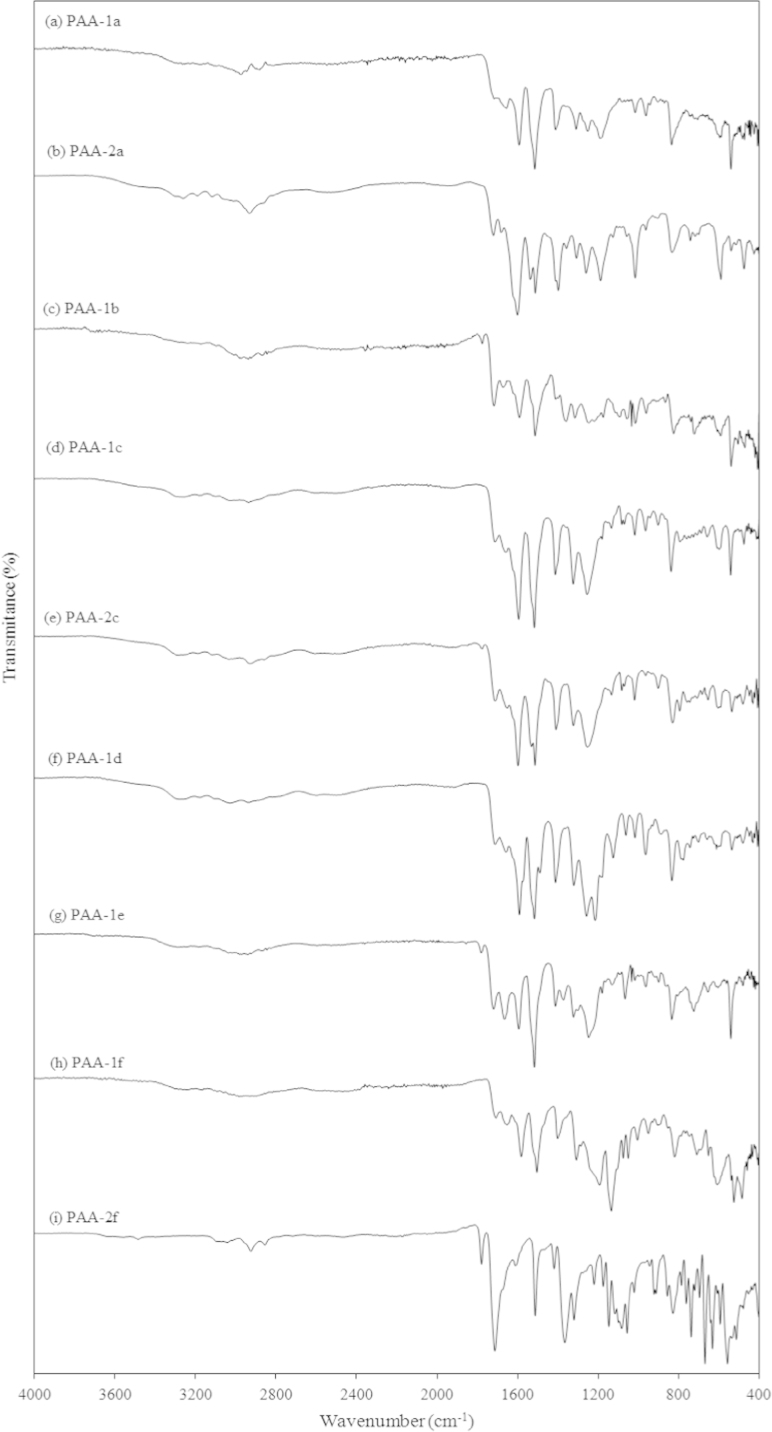
FT-IR spectra of (a) PAA-1a, (b) PAA-2a, (c) PAA-1b, (d) PAA-1c, (e) PAA-2c, (f) PAA-1d, (g) PAA-1e, (h) PAA-1f and (i) PAA-2f.

**Fig. 3:**
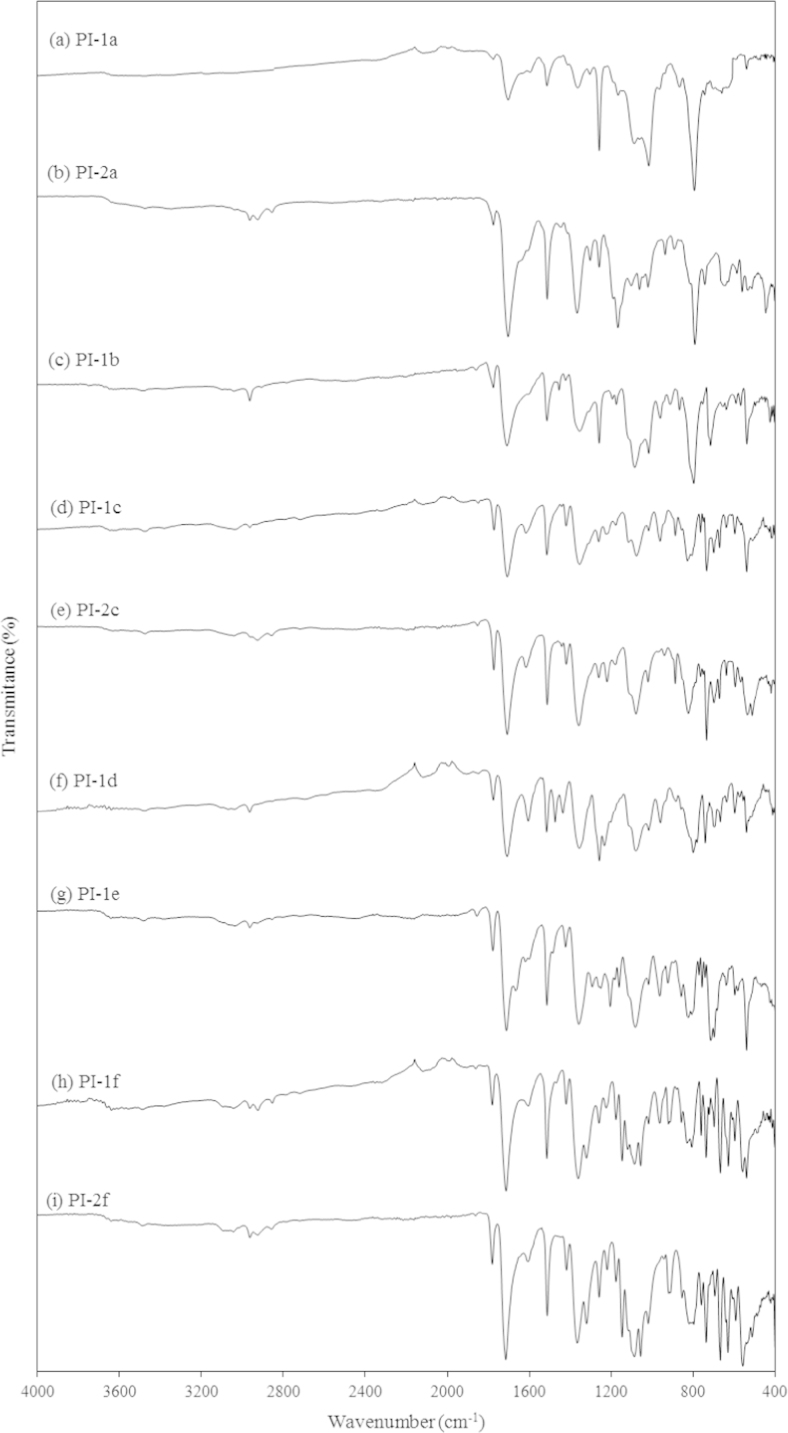
FT-IR spectra of (a) PI-1a, (b) PI-2a, (c) PI-1b, (d) PI-1c, (e) PI-2c, (f) PI-1d, (g) PI-1e, (h) PI-1f and (i) PI-2f.

Fig. 2, Fig. 3 show the FT-IR spectra of the PAAs and PIs respectively. In all the samples the following peaks were observed: broad band in the range 2500–3500 cm^−1^ (O–H stretching of carboxylic acid group’s hydroxyls), two different carbonyl peaks at 1720 cm^−1^ (C=O stretching, carboxylic) and 1670 cm^−1^ (C=O stretching, amide), and 1520 and 1430 cm^−1^ (aromatic C–H overtone aromatic). After imidization 1720 cm^−1^ (C=O stretching, carboxylic) and 1670 cm^−1^ (C=O stretching, amide) were replaced by a small peak at 1780 cm^−1^ (C=O asymmetric stretching) and a big peak at 1712 cm^−1^ (C=O symmetric stretching) which were characteristic to PI structures. Furthermore, other peaks at 1518 cm^−1^ (C–C stretching of aromatic), 1441 cm^−1^ (C=C stretching of p-substituted benzene) 1376 cm^−1^, (C–N stretching of imide), and 1175 cm^−1^ (imide ring deformation) appeared, which indicated a complete imidization [1]. OPDA derived PI-1d showed IR peak at 1235 cm^−1^ corresponding to C–O stretching of ether group and DSPDA derived PI-1f and PI-2f showed asymmetric and symmetric S=O stretching at 1323 and 1148 cm^−1^ respectively [2].

## 4 Experimental design, materials and methods

For NMR DMSO-*d*_6_ was used as a solvent at 23.1 °C with 16 accumulation scans, using the proton resonance of residual non-deuterated DMSO as an internal standard (2.55 ppm). PAAs and PIs film of thinkness 30–45 µm was used for FT-IR analysis. PAA film of thickness 30–45 µm was obtained by dissolving polymer fibrils in DMAc/DMF forming a yellow solution, poured on a silicon wafer/aluminum plate by spin coating and dried by heating over hot plate. Polyimide (PI) films were obtained by stepwise thermal imidization of the PAAs film in an oven for 1 h each at 100, 150, 200 and 250 °C, respectively, under vacuum [3].
